# In Vitro Assessment of Gold Nanoparticles Synthesized by Gamma Irradiation for Antimicrobial and Anticancer Effects

**DOI:** 10.3390/microorganisms13112424

**Published:** 2025-10-23

**Authors:** Doaa E. El-Hadedy, Nesreen A. Safwat, Hoda H. Saleh, Zakaria I. Ali

**Affiliations:** 1 Radiation Microbiology Department, National Center for Radiation Research and Technology, Egyptian Atomic Energy Authority, Cairo 11787, Egypt; 2 Kornberg Dentistry School, Temple University, Philadelphia, PA 19140, USA; 3 The Regional Center for Mycology and Biotechnology, Al-Azhar University, Cairo 11884, Egypt; 4 Radiation Chemistry Department, National Center for Radiation Research and Technology, Egyptian Atomic Energy Authority, Cairo 11787, Egypt

**Keywords:** gold nanoparticles, antibacterial, antiviral, anticancer activity

## Abstract

The emergence of multidrug-resistant microbes presents a serious public health threat that requires new antimicrobial methods. A potential solution to combat resistance involves using metal nanoparticles that possess improved biological characteristics. The researchers have synthesized gold nanoparticles (Au-NPs) using gamma irradiation of Polyacrylamide (PAM) at 5, 10, and 15 kGy doses and through Au/chitosan nanocomposite production methods. They have also assessed the antimicrobial and anticancer functions of the produced nanomaterials by testing them on various microorganisms and cancer cell lines. Gold nanoparticles exhibited strong antibacterial effects against multiple Gram-positive bacterial strains, including *Bacillus subtilis*, *Micrococcus luteus*, *Staphylococcus epidermidis*, *Staphylococcus aureus*, *and Streptococcus mutans*, as well as methicillin-resistant *Staphylococcus aureus* (MRSA). *Escherichia coli* showed a significant inhibition zone of 23 mm, and *Salmonella* spp. showed similar inhibition. The inhibition zone for *Klebsiella pneumoniae* ATCC 13883 revealed resistance. The Au-NPs/chitosan composite showed moderate antifungal effectiveness against *Syncephalis racemosum* and *Aspergillus niger* alongside *Candida albicans* and several other tested fungi. Au-NPs showed cytotoxicity to breast MCF-7 cells, as well as liver HepG-2 cells and colon HCT-116 cells. The combination of Au-NPs with chitosan demonstrated limited effectiveness in countering hepatitis A virus (HAV-10) and herpes simplex virus type 1 (HSV-1). The combination of gamma-irradiated Au-NPs with biopolymers like chitosan demonstrates significant promise in antimicrobial and anticancer biomedical applications.

## 1. Introduction

Nanotechnology has enabled the precise control and manipulation of nanostructured materials, endowing them with unique physicochemical properties due to their small size. As the surface area of metal nanoparticles (NPs) increases, they exhibit distinctive physical, chemical, and biological characteristics. The synthesis of metal NPs has garnered significant attention in recent years, as they offer promising opportunities for diverse technical and biomedical applications [1,2].

Gold nanoparticles (Au-NPs) have emerged as a particularly exciting focus of research due to their remarkable optical, electronic, magnetic, pharmaceutical, and medical properties [3]. Among the various functional polymers used for assembling metal nanoparticles, chitosan and polyacrylamide offer notable advantages. These polymers are water-soluble, biocompatible, and biodegradable, with no reported toxicity. Moreover, their physicochemical and biological properties make them suitable for biomedical applications, including anti-inflammatory, antioxidant, antihypertensive, anticancer, and antidiabetic therapies [4].

In recent years, nanotechnology has gained considerable traction in medical research, leading to the development of nanoparticles as effective cancer cell detectors. The radioactive, optical, or magnetic properties of certain nanoparticles facilitate early tumor imaging, while others can be functionalized as anticancer nanocarriers for targeted drug delivery. The emergence of nanoparticles as theranostic agents—combining therapeutic and diagnostic functions—is expected to revolutionize cancer management [5].

Early research on nanoparticle–microbe interactions demonstrated that cationic gold nanoparticles exhibit antibacterial toxicity [6]. Further studies revealed that hydrophobic, cationic Au-NPs formed distinct spatiotemporal aggregate patterns on bacterial surfaces, varying by bacterial type and NP size [7]. Additionally, Refs. [8,9] confirmed that nanoparticle surface chemistry plays a critical role in NP–bacteria interactions. They observed that highly cationic NPs had the strongest affinity for bacterial surfaces, resulting in significant membrane disruption and toxicity. These findings provide valuable insights for designing nanoparticle-based antibacterial therapies.

In this study, the biological activities of PAM/Au nanocomposites (irradiated at 5, 10, and 15 kGy) and Au/chitosan nanocomposites were investigated to evaluate their potential as antimicrobial and anticancer agents.

## 2. Materials and Methods

### 2.1. Chemicals Used

Dimethyl sulfoxide (DMSO), MTT, and trypan blue dye were purchased from Sigma (St. Louis, MO, USA). Fetal Bovine serum, DMEM, RPMI-1640, HEPES buffer solution, L-glutamine, gentamycin, and 0.25% Trypsin-EDTA were purchased from Lonza, Belgium. Polyacrylamide (PAM), a white granular powder with a molecular weight of approximately 5,000,000 g/mol, was purchased from Sigma-Aldrich. Chitosan (Cs), a deacetylated form of chitin [poly(D-glucosamine), (C_6_H_11_NO_4_)_n_], with a viscosity of 50–300 and purity >90%, was also obtained from Sigma-Aldrich. Gold chloride hydrate [HAuCl_4_·H_2_O], with a formula weight of 357.79, was purchased from Electron Microscopy Sciences.

### 2.2. Mammalian Cell Lines

Vero cells (African green monkey kidney cell line), HepG-2 (human hepatocellular carcinoma), MCF-7 (human breast carcinoma), and HCT-116 (human colon carcinoma) cell lines were obtained from the American Type Culture Collection (ATCC, Rockville, MD, USA). Viral isolates, including Herpes simplex virus type 1 (HSV-1) and Hepatitis A virus type 10, were purchased from the Holding Company for Biological Products and Vaccines (VACSERA), Giza, Egypt. Viruses were propagated and titrated on Vero cells and stored in aliquots at −70 °C until use.

### 2.3. Preparation of PAM/Au and Chitosan/Au Nanoparticles

Polyacrylamide (PAM) and chitosan (Cs) were each dissolved in 100 mL of deionized water at 1 wt% and stirred for 4 h at room temperature until fully dissolved. One milliliter of 0.025 M HAuCl_4_ was then added to 10 mL of each polymer solution to prepare PAM/Au and Cs/Au colloidal nanoparticles. The Au-NP concentration for biological assays was calculated based on the known molarity of the gold precursor (0.025 M HAuCl_4_) in 1% polymer solutions [10]. UV–Vis spectrophotometry confirmed successful nanoparticle formation via surface plasmon resonance (SPR) peaks at 530–570 nm, consistent with Au-NP sizes of 10–30 nm [3,11].

### 2.4. Irradiation Process

PAM/Au and Cs/Au solutions were irradiated with gamma rays at doses of 5, 10, and 15 kGy using a Co-60 γ-cell-220 source (Egyptian Atomic Energy Authority), installed at the National Center for Radiation Research and Technology (NCRRT), Egyptian Atomic Energy Authority (EAEA), Nasr City, Cairo, Egypt. These doses were selected based on prior optimization studies supporting efficient nucleation and colloidal stability [11].

### 2.5. Antibacterial Activity of Au-NPs

Antibacterial activity was assessed using the agar well diffusion method. Gram-positive strains tested included *Bacillus subtilis* (RCMB 015), *Micrococcus luteus* (RCMB 018), *Staphylococcus epidermidis* (RCMB 009), MRSA, *Staphylococcus aureus* (RCMB 007), and *Streptococcus mutans* (RCMB 017). Gram-negative strains included *Salmonella* spp. (RCMB 010043), *Escherichia coli* (RCMB 010052), *Enterobacter cloacae* RCMB 001(1) ATCC 23355, *Klebsiella pneumoniae* RCMB 003(1) ATCC 13883, *Proteus vulgaris* RCMB 004(1) ATCC 13315, and *Salmonella typhimurium* RCMB 006(1) ATCC 14028.

Briefly, 100 µL of each bacterial suspension (~10^8^ CFU/mL) was inoculated into Mueller–Hinton agar plates. Wells (6 mm diameter) were filled with 100 µL of each Au-NP sample (1 molar). Plates were incubated at 37 °C for 24 h, and inhibition zones were measured in millimeters. The minimum inhibitory concentration (MIC) was also determined for Au/chitosan, the most effective formulation [12].

### 2.6. Antifungal Activity of Au-NPs

Antifungal activity was evaluated against various fungal strains using the agar well diffusion method. Test strains included *Aspergillus fumigatus* (RCMB 02568), *Candida albicans* (RCMB 05036), *Aspergillus niger* (RCMB 002005), *Aspergillus flavus* (RCMB 002002), *Penicillium aurantiogriseum* IMI 89372, and *Syncephalis racemosum* (RCMB 016001). Fungal inoculum (10⁵ cells/mL) was prepared, and 100 µL of Au-NP samples (1 molar) was added to each well. Plates were incubated at 37 °C for 24–48 h (yeasts) or 28 °C for 48 h (filamentous fungi), and inhibition zones were measured in triplicate [13].

### 2.7. Anticancer Activity of Au-NPs (MTT Assay)

HepG2, MCF-7, and HCT-116 cells were cultured in RPMI-1640 supplemented with 10% fetal bovine serum and 50 µg/mL gentamycin, maintained at 37 °C and 5% CO_2_. Cells (5 × 10^4^/well) were seeded into 96-well plates and incubated for 24 h. Au-NP samples were added at six different concentrations (in triplicate), and cells were incubated for another 24 h. MTT reagent (10 µL of 12 mM stock) was added to each well with 100 µL of fresh medium. After 4 h, 85 µL of medium was removed, and 50 µL of DMSO was added. Absorbance was measured at 590 nm using a microplate reader (SunRise, TECAN, Morris Plains, NJ, USA). Cell viability was calculated as:

Viability (%) = (OD_t_/ODc) × 100

where OD_t_ = treated cells, and ODc = untreated control. IC_50_ values were calculated using GraphPad Prism version 10.3.1, which was released on 28 August 2024 [14].

### 2.8. Antiviral Activity of Au-NPs

Antiviral activity was evaluated using the cytopathic effect (CPE) inhibition assay. Vero cells (1 × 10^4^ cells/well) were seeded into 96-well plates and incubated for 24 h at 37 °C with 5% CO_2_. Wells were washed and infected with 10^4^ viral doses of HSV-1 or HAV-10. Non-toxic concentrations of Au-NPs were added simultaneously. Control groups included virus-infected untreated cells and untreated uninfected cells. CPE was monitored daily using an inverted microscope. Antiviral activity was assessed by comparing CPE inhibition in treated vs. control wells [15].

### 2.9. Statistical Analysis

All experiments were performed in triplicate (*n* = 3), and the results are presented as mean ± standard deviation (SD). Statistical analysis was conducted using one-way ANOVA followed by Tukey’s post hoc test. A *p*-value < 0.05 was considered statistically significant [16]. Standard deviation and significance were calculated using Microsoft Excel and GraphPad Prism.

## 3. Results and Discussion

The UV–Visible (UV/VIS) spectrophotometer analyzed the synthesized chitosan/gold (CS/Au) and polyacrylamide/gold (PAM/Au) colloidal nanoparticles across the 200–800 nm wavelength spectrum. The CS/Au samples displayed a surface plasmon resonance (SPR) peak from 530 to 550 nm, while the PAM/Au samples exhibited an SPR peak near 570 nm. Several factors, including polymer matrix type and nanoparticle content, together with shape, size, and size distribution, influence the variations in SPR peak position. The UV/VIS analysis results demonstrated that the SPR peak intensity for CS/Au samples rose when irradiation doses increased. The peaks showed increased sharpness at higher doses, which demonstrated enhanced nanoparticle formation and improved dispersion throughout the polymer matrix.

The superior antimicrobial and anticancer effects seen in chitosan-based Au-NPs when compared to PAM-based formulations likely result from distinct physicochemical property differences. Chitosan serves as a naturally occurring cationic polysaccharide that displays both biocompatibility and biodegradability while possessing natural antimicrobial properties. Chitosan’s positive surface charge facilitates powerful electrostatic bond formation with negatively charged microbial and cancer cell membranes, which results in greater nanoparticle absorption and cellular damage [14,17]. PAM represents a synthetic non-ionic polymer with no innate biological activity, which produces a stable but inactive layer on nanoparticles that might restrict their cellular interactions and absorption. The observed reduction in biological activity of PAM-based nanocomposites in multiple assays can be explained by their structural and functional differences.

**Figure 1:**
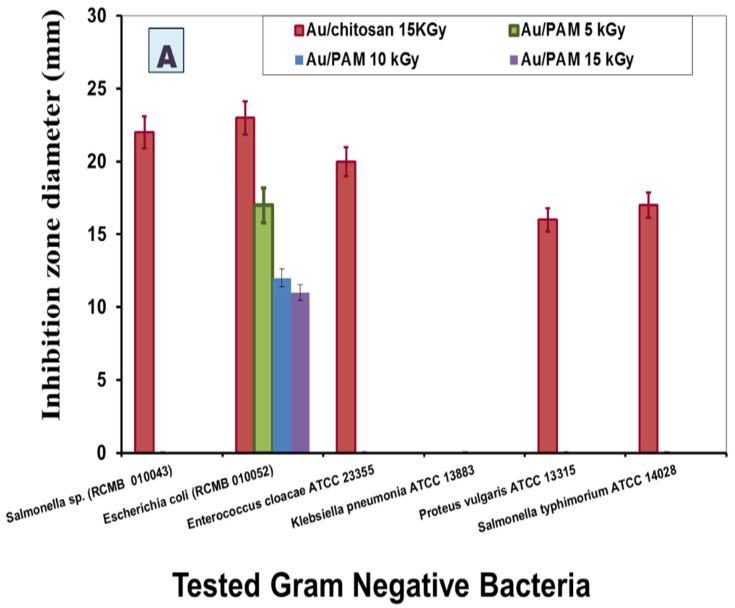

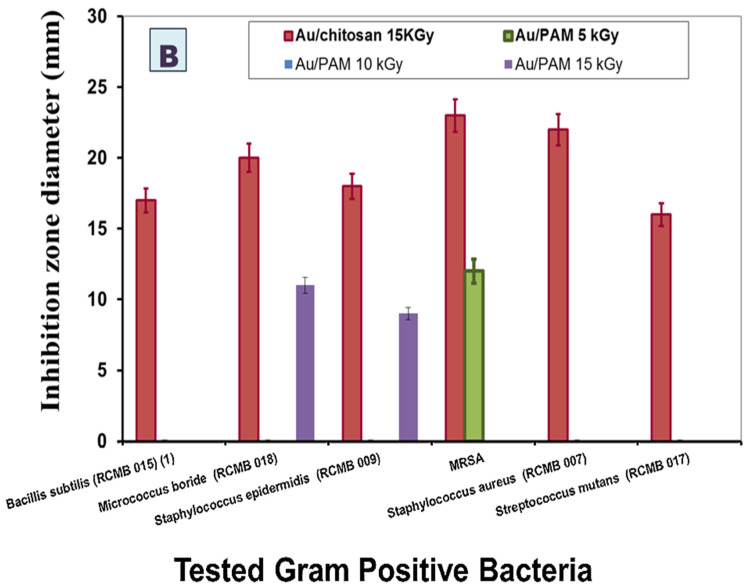
The antibacterial activity of the tested gold nanoparticles is expressed as the mean inhibition zone diameter (in mm) against a range of clinically relevant Gram-negative (**A**) and Gram-positive (**B**) bacteria, using the agar diffusion technique. Data represent mean ± SD of three independent experiments (*n* = 3). Statistical significance was determined by one-way ANOVA with Tukey’s post hoc test (*p* < 0.05).

Researchers assessed gold nanoparticles’ antibacterial effects by determining the average size of inhibition zones in millimeters and the minimal inhibitory concentration in micrograms per milliliter needed to stop bacterial growth. Figure 1 demonstrates that Au–chitosan nanoparticles have strong antibacterial properties, but Au-PAM nanoparticles irradiated at dosages of 5, 10, and 15 kGy do not show any antimicrobial effectiveness against Bacillus subtilis (RCMB 015), *Micrococcus luteus* (RCMB 018), *Staphylococcus epidermidis* (RCMB 009), methicillin-resistant *Staphylococcus aureus* (MRSA), *Staphylococcus aureus* (RCMB 007), and *Streptococcus mutans* (RCMB 017). This study investigated nanoparticle formation and biological activity through the application of radiation doses at 5 kGy, 10 kGy, and 15 kGy. This study’s results match previous research findings by demonstrating that increased irradiation doses elevate nanoparticle production, which leads to enhanced antimicrobial and anticancer effects [11].

The measurement of inhibition zone diameters in agar diffusion tests cannot serve as direct measures of MIC values in nanoparticle-based systems. Zone size can be affected by molecular weight and viscosity of chitosan as well as nanoparticle–polymer interactions and diffusion constraints in the agar matrix, regardless of the actual antimicrobial potency of the nanoparticles [8,17].

**Scheme 1:**
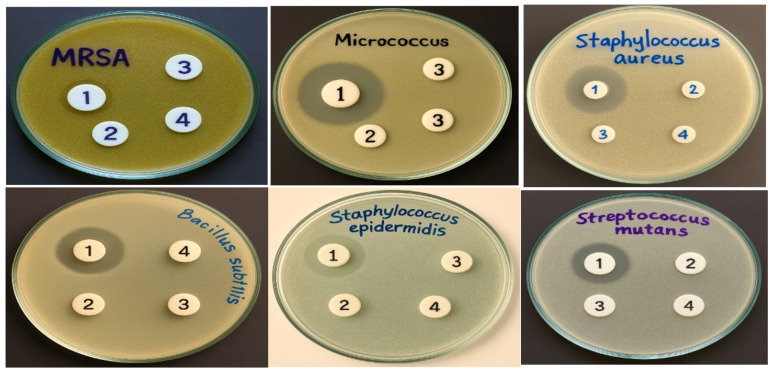
The susceptibility of some gram-positive bacteria when treated with Au-NPs was assessed using the agar well diffusion method [samples 1, 2, and 3: Au-PAM at a dose of 15, 10, and 5 KGy, respectively; sample 4: Au–chitosan at 15 KGy]. *Staphylococcus aureus* (RCMB 007), *Micrococcus boride* (RCMB 018), MRSA, *Bacillis subtilis* (RCMB 015), *Staphylococcus epidermidis* (RCMB 009), and *Streptococcus mutans* (RCMB 017).

**Scheme 2:**
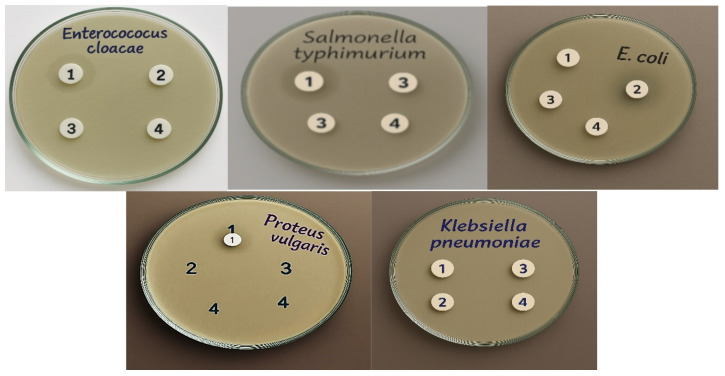
The susceptibility of some Gram-negative bacteria when treated with Au-NPs was assessed using the agar well diffusion method [samples 1, 2, and 3: Au-PAM at a dose of 15, 10, and 5 KGy, respectively; sample 4: Au–chitosan at 15 KGy. *Enterococcus cloaca* ATCC 23355, *Escherichia coli* (RCMB 010052), *Klebsiella pneumonia* ATCC 13883, *Salmonella typhimorium* ATCC 14028, and *Proteus vulgaris* ATCC 13315.

The antibacterial ability of Au–chitosan nanoparticles was particularly effective against MRSA and Staphylococcus aureus (RCMB 007), as evidenced by mean inhibition zones of 23 mm and 22 mm, respectively. The antibacterial actions of Au–chitosan nanoparticles against Gram-negative bacteria such as *Salmonella* sp. were demonstrated in Scheme 1 and Scheme 2. The Au–chitosan nanoparticles showed effective antibacterial action against Gram-negative bacteria such as *Salmonella* sp. (RCMB 010043), Escherichia coli (RCMB 010052), *Enterobacter cloacae* (RCMB 001(1) ATCC 23355), *Klebsiella pneumoniae* (RCMB 003(1) ATCC 13883), *Proteus vulgaris* (RCMB 004(1) ATCC 13315), and *Salmonella typhimurium* (RCMB 006(1) ATCC 14028). Au–chitosan nanocomposites demonstrate significant potential for use in antimicrobial applications.

On the other hand, our results demonstrated that the MIC of Au–chitosan NPs against the tested Gram-positive and Gram-negative bacterial strains ranged from 63 to 250 µM, along with a weak effect on the tested *Klebsiella pneumonia* strain (Table 1).

### 3.1. Antifungal Activity

**Figure 2:**
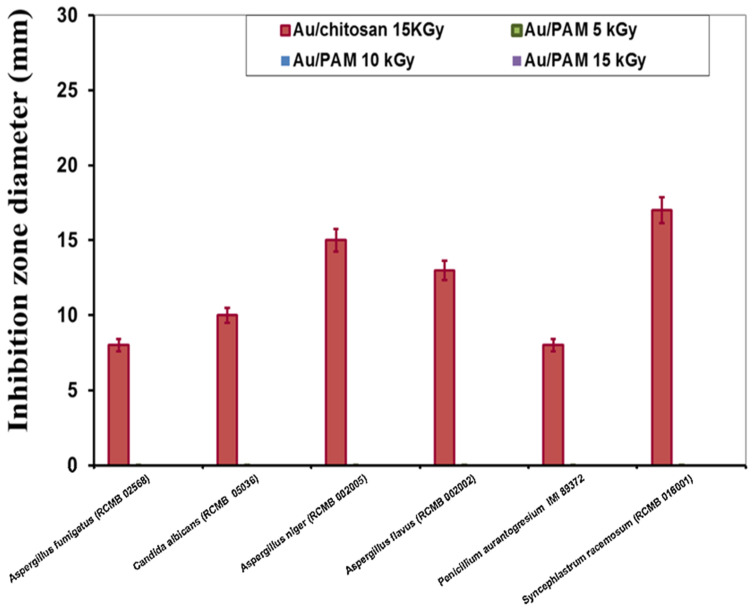
The antifungal activities of the tested Au/Chitosan nanoparticles expressed as the mean zone of inhibition (in mm), tested using the agar diffusion technique. Data represent mean ± SD of three independent experiments (*n* = 3). Statistical significance was determined by one-way ANOVA with Tukey’s post hoc test (*p* < 0.05).

In the current study, Au-NPs were tested as an antifungal agent against *Aspergillus fumigatus* (RCMB 02568), *Candida albicans* (RCMB 05036), *Aspergillus niger* (RCMB 002005), *Aspergillus flavus* (RCMB 002002), *Penicillium auragtogresium* IMI 89372, and *Syncephlastrum racemosum* RCMB 016001. The antifungal activities of Au–chitosan nanoparticles and Au–PAM nanoparticles at three doses of gamma radiation (5, 10, and 15 kGy) were examined on the basis of inhibition zone and are reported in Figure 2.

Different compounds of Au-NPs showed various activities towards different fungal strains, with inhibition areas ranging from 8 mm to 17 mm after treatment with Au–chitosan nanoparticles. However, treatment with Au–PAM nanoparticles at all radiation doses exhibited no inhibition in any of the six tested fungal strains. The difference in the diameter of the zone inhibition areas may be attributed to the difference in the sensitivity of the tested strains to Au–chitosan NPs. Data illustrate that the most sensitive fungus to Au–chitosan nanoparticles treatment is *Syncephlastrum racemosum*, with measured minimum inhibition concentration of 125 µM, while the most resistant ones are *Aspergillus fumigatus* (RCMB 02568) and *P. aurantogresium* IMI 89372, because both showed an MIC value of 1000 µM (Table 2).

This study found that fungal species show variable sensitivity levels because of differences in their cell wall composition, membrane structure, and physiological characteristics. Antifungal effects of chitosan-based gold nanoparticles happen through their interaction with the negative charge of fungal cell surfaces, which leads to membrane damage and oxidative stress generation. Species variations determine the degree of interactions between cells and nanoparticles. Fungi that possess high levels of chitin or β-glucan in their structure tend to resist nanoparticles better because these components decrease the penetration or binding of the particles. The plasma membrane’s susceptibility to nanoparticle-induced damage varies depending on its ergosterol levels and lipid composition. Our observations agree with earlier studies that demonstrate species-specific effects from chitosan and nanoparticle treatments [17,18]. The antifungal activity differences noted in this study probably result from the unique structural and biochemical properties inherent to each fungal strain.

### 3.2. Anti-Cancer Activity

This study aimed to evaluate the anticancer potential of gold nanoparticles (Au-NPs) against three cancer cell lines: MCF-7 (breast cancer), HepG-2 (hepatocellular carcinoma), and HCT-116 (colon carcinoma). The cells were treated with Au–PAM nanoparticles synthesized with three different gamma radiation doses (5, 10, and 15 kGy) and Au–chitosan nanoparticles for 24 h. Following exposure, cell viability was assessed using the MTT assay.

**Figure 3:**
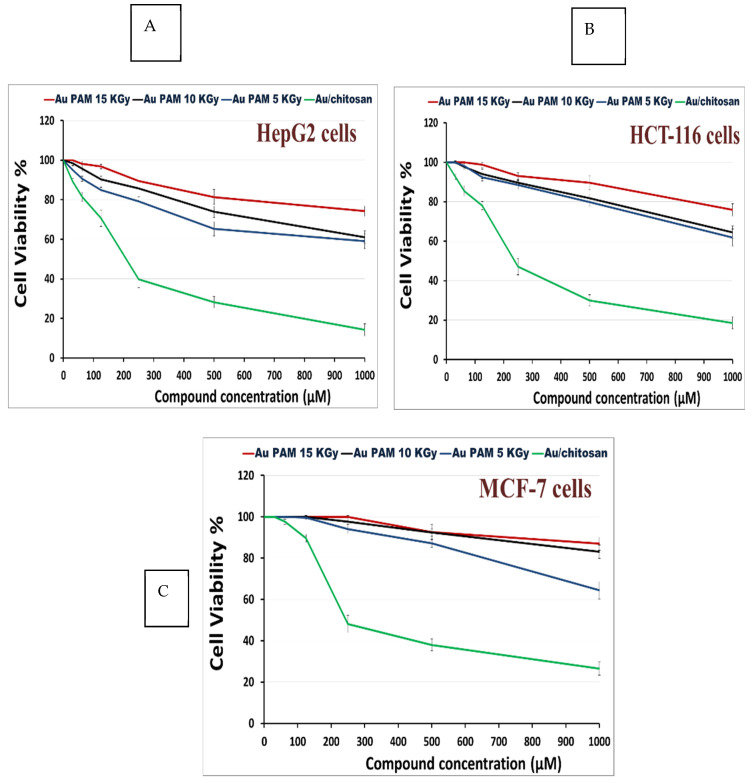
(**A**) The inhibitory activities of the tested gold nanoparticles against the hepatocellular carcinoma (HepG2) cell line expressed in dose response curves as remaining viable cells after exposure to different concentrations of nanoparticles, measured using the MTT assay. (**B**) The inhibitory activities of the tested gold nanoparticles against the colon carcinoma (HCT-116) cell line expressed in dose–response curves as remaining viable cells after exposure to different concentrations of nanoparticles, measured using the MTT assay. (**C**) The inhibitory activities of the tested gold nanoparticles against the breast carcinoma (MCF-7) cell line expressed in dose response curves as remaining viable cells after exposure to different concentrations of nanoparticles, measured using the MTT assay. Data represent mean ± SD of three independent experiments (*n* = 3). Statistical significance was determined by one-way ANOVA with Tukey’s post hoc test (*p* < 0.05).

As illustrated in Figure 3A–C, a significant decrease in cell viability was observed with increasing concentrations of Au-NPs. In the case of the HepG-2 cell line, Au–chitosan nanoparticles exhibited strong inhibitory activity against hepatocellular carcinoma cells, with an IC_50_ value of 208.7 µM (Figure 3A). Similarly, Au–chitosan nanoparticles effectively inhibited the growth of HCT-116 colon carcinoma cells, with an IC_50_ value of 238.9 µM (Figure 3B). Additionally, significant inhibition was observed in MCF-7 breast cancer cells, with an IC_50_ value of 244 µM (Figure 3C). Conversely, Au–PAM nanoparticles showed no significant inhibitory activity against the three cancer cell lines, with IC_50_ values exceeding 1000 µM. These findings suggest that Au–chitosan nanoparticles exhibit promising anticancer effects, whereas Au–PAM nanoparticles demonstrate limited cytotoxicity against the tested cancer cell lines.

The current study focused solely on cancer cells using an MTT assay for three cancer lines (MCF-7, HepG2, and HCT-116) to analyze the anticancer capabilities of synthesized Au-NPs without evaluating cytotoxic effects on normal cell lines. Research indicates that gold nanoparticles stabilized with biocompatible polymers like chitosan show diminished cytotoxic effects on normal cells because of their decreased cellular uptake and reduced oxidative stress production [14,19,20]. Chitosan enhances selectivity by promoting stronger interactions with cancer cells, which exhibit higher metabolic activity and more negatively charged membranes. The current research results demonstrate anticancer activity that matches selective cytotoxicity outcomes from previous studies but requires additional validation through testing on normal epithelial or fibroblast cell lines in future work.

### 3.3. Antiviral Activity

This study presents the first report on the antiviral activity of gold nanoparticles against HSV-1 and HAV-10 (Table 3). Nanotechnology has enabled the precise control and manipulation of nanostructured materials, introducing novel physicochemical properties due to their nanoscale dimensions. As the surface area of metal nanoparticles increases, their unique physical, chemical, and biological properties become more pronounced.

The surface plasmon resonance (SPR) peak for CS/Au samples exhibited a shift towards lower wavelengths with increasing irradiation dose, known as a blue shift. This shift corresponds to a reduction in nanoparticle size. In contrast, the irradiated CS/Au samples displayed a shoulder SPR peak centered at approximately 570 nm, which suffered from significant distortion and broadening, accompanied by a long absorption tail. This observation suggests that the gold nanoparticles in these samples exhibit a broad size distribution and are larger than those in PAM/Au samples. The data indicate that a 10 kGy irradiation dose is optimal for synthesizing gold colloidal nanoparticles, as it resulted in the highest SPR intensity [3,11].

Bacterial infections remain a major global health concern, causing approximately 300 million cases of severe illness annually, with 16 million fatalities, including 2 million children [21]. The emergence of multidrug-resistant (MDR) bacteria further exacerbates this issue, posing a significant challenge to infection control. Beyond acute illness, bacterial infections can lead to chronic disease states, where bacterial colonization evolves into biofilms—complex, three-dimensional bacterial communities that are notoriously difficult to eradicate [22,23,24]. 

Several reports demonstrated that Au-NPs have antibacterial activity against a wide range of positive and negative bacteria. Nanoparticles (NPs) emerge as weapons in our antimicrobial arsenal owing to their unique nanoscale physical and chemical properties [25,26]. Nanoparticles (NPs) possess sizes comparable to biomolecules and bacterial cellular systems, allowing for precise tuning of nanomaterial-bacteria interactions through appropriate surface functionalization [10,27]. Additionally, their high surface area-to-volume ratio enables a substantial therapeutic payload, with multivalent interactions enhancing their antimicrobial effectiveness. NPs present a viable strategy to counteract common antibiotic resistance mechanisms, including permeability regulation [28,29], multidrug efflux pumps [30], and mutations that alter target site binding affinity [31]. Moreover, NPs offer alternative pathways to combat biofilm-associated and multidrug-resistant (MDR) infections while significantly reducing the likelihood of bacterial resistance over time [32,33]. By utilizing multiple bacterial-killing mechanisms, NPs make it difficult for pathogens to adapt existing resistance strategies [34]. Consequently, various NP-based systems have been developed to enhance antimicrobial efficacy.

Chitosan, a biocompatible polymer derived from crustacean exoskeletons, provides a versatile platform for designing antimicrobial coatings. A study using a central venous catheter model demonstrated that chitosan-coated catheters effectively inhibited *Candida albicans* biofilm formation in vivo [18]. Chitosan significantly reduced the metabolic activity of *Candida parapsilosis* biofilms and decreased the cell viability of *Cryptococcus neoformans* [18,34]. Notably, melanization, a key virulence factor of *C. neoformans*, did not protect cryptococcal biofilms from chitosan treatment [35]. This phenomenon is attributed to the ability of cationic chitosan to disrupt negatively charged microbial membranes during surface colonization [17]. By altering the fungal cell membrane’s negative charge, chitosan interferes with microbial adhesion, cell–cell interactions, and biofilm formation [36].

Furthermore, gold nanoparticles (Au-NPs) have demonstrated antiviral activity against both DNA and RNA viruses, including herpes simplex virus (HSV), human parainfluenza virus type 3, H3N2 influenza virus, vaccinia virus, and adenovirus type 3 [36,37,38,39]. Recent studies have shown that Au-NPs can reduce the cytopathic effect of HSV-1 in Vero cells in a dose- and time-dependent manner, suggesting their potential as antiviral agents. Additionally, Au-NPs have been reported to exhibit antiviral effects against the influenza A virus [40]. The limited antiviral action from PAM–Au-NP formulations could result from the diminished binding between PAM-coated nanoparticles and viral envelopes or cellular membranes of host cells. PAM does not possess intrinsic bioactivity or electrostatic affinity like chitosan does, which could restrict nanoparticle uptake and membrane disruption necessary for antiviral effectiveness.

The exploration of nanoparticle-based antiviral drugs for Herpes Simplex Virus (HSV) has emerged as a recent research focus, with only a limited number of structures proposed. [41] investigated the antiviral potential of silver (Ag) and gold (Au) nanoparticles functionalized with sulfonate groups, synthesized via a sonochemical approach, for the inhibition of HSV-1 infection [41,42]. Consistent with our findings, mercaptoethanesulfonate (MES)-functionalized Ag NPs, approximately 4 nm in size, exhibited a heparan sulfate (HS)-mimicking effect at a concentration of 40 µg/mL. These nanoparticles effectively blocked viral attachment, inhibited viral entry into host cells, and prevented cell-to-cell transmission of HSV-1 [42]. 

The results align with new research that shows that gold nanoparticles made from natural polymers exhibit improved antimicrobial and anticancer activity. For example, [43]. The study from 2020 confirmed that chitosan-stabilized gold nanoparticles showed significant antibacterial activity against both Gram-positive and Gram-negative bacteria, such as *S. aureus* and *E. coli*, with comparable inhibition zones [43]; Similarly, [44] mentioned the cytotoxicity research conducted by [44] demonstrated that biopolymer-based Au-NPs were effective against MCF-7 and HepG-2 cell lines, which agrees with our findings on dose-dependent anticancer effects [44]. A study conducted by [45] revealed that alginate-coated Au-NPs demonstrated minimal antifungal activity against *C. albicans*. The investigation by [45] revealed that alginate-coated Au-NPs demonstrated only minimal antifungal effects against *C. albicans* which matches our findings of limited effectiveness against fungal strains [45]. The comparative studies emphasize how nanoparticle synthesis conditions, together with polymer selection, play crucial roles in determining biological outcomes.

## 4. Conclusions

The antimicrobial and anticancer activities of gold nanoparticles (Au-NPs) were thoroughly investigated. Gold nanoparticles were synthesized in situ via a gamma-irradiation process using Polyacrylamide (PAM) at doses of 5, 10, and 15 kGy, as well as Au/chitosan nanocomposites. The Au-NPs exhibited strong antibacterial effects against various Gram-positive bacteria, including *Bacillus subtilis*, *Micrococcus boride*, *Staphylococcus epidermidis*, *Staphylococcus aureu*s, *Streptococcus mutans*, and notably, Methicillin-resistant *Staphylococcus aureus* (MRSA). Similarly, the tested Au-NPs also inhibited several Gram-negative bacteria, with significant antibacterial effects observed against *Escherichia coli* (23 mm) and *Salmonella* sp. (22 mm), compared to reference standard antibacterial drugs. However, no activity was observed against *Klebsiella pneumoniae* ATCC 13883. Furthermore, Au-NPs with chitosan showed moderate activity against fungi, with the highest efficacy being against *Syncephlastrum racemosum*, followed by *Aspergillus niger*, *Aspergillus flavus*, *Candida albicans*, *Penicillium aurantogresium*, and *Aspergillus fumigatus.* Additionally, Au-NPs demonstrated significant cytotoxic potential against three cancer cell lines: MCF-7, HepG-2, and HCT-116. The synthesized Au-NPs with chitosan also showed weak antiviral activity against HAV-10 and HSV-1. These findings suggest that Au-NPs could be developed as effective antimicrobial and anticancer agents. The results underscore the need for innovative strategies to enhance the efficacy of drugs in combating emerging infectious diseases and microbial resistance.

This study presents several limitations, such as a lack of normal cell line cytotoxicity data and in vivo validation. Subsequent research will target dose–response optimization and assess both therapeutic effectiveness and safety of these nanocomposites through animal studies.

## Figures and Tables

**Table 1:** Minimum inhibitory concentration (MIC) for Au/chitosan nanoparticles determined against the tested bacteria using the diffusion agar technique.

Tested Gram-Positive Bacteria	MIC (µM)	Tested Gram-Negative Bacteria	MIC (µM)
*Bacillis subtilis* (RCMB 015) (1)	250	*Salmonella* sp. (RCMB 010043)	125
*Micrococcus boride* (RCMB 018)	125	*Escherichia coli* (RCMB 010052)	63
*Staphylococcus epidermidis* (RCMB 009)	63	*Enterococcus cloaca* ATCC 23355	125
*MRSA*	250	*Klebsiella pneumonia* ATCC 13883	undetermined
*Staphylococcus aureus* (RCMB 007)	125	*Proteus vulgaris* ATCC 13315	250
*Streptococcus mutans* (RCMB 017)	125	*Salmonella typhimorium* ATCC 4028	125

Data represent mean ± SD of three independent experiments (*n* = 3). Statistical significance was determined by one-way ANOVA with Tukey’s post hoc test (*p* < 0.05).

**Table 2:** Minimum inhibitory concentration for Au/chitosan nanoparticles against the tested pathogenic fungi.

Tested Fungi	Au/Chitosan (MIC; µM)
*Aspergillus fumigatus* (RCMB 02568)	1000
*Candida albicans* (RCMB 05036)	500
*Aspergillus niger* (RCMB 002005)	250
*Aspergillus flavus* (RCMB 002002)	500
*Penicillium aurantogresium* IMI 89372	1000
*Syncephlastrum racemosum* (RCMB 016001)	125

Data represent mean ± SD of three independent experiments (*n* = 3). Statistical significance was determined by one-way ANOVA with Tukey’s post hoc test (*p* < 0.05).

**Table 3:** The antiviral effect of gold nanoparticles on HSV-1 and HAV-10 tested at 1 molar.

Gold Nanoparticles	Antiviral Effect On	
	HSV-1	HAV-10
Au/PAM (15 KGy)	-ve	-ve
Au/PAM (10 KGy)	-ve	-ve
Au/PAM (5 KGy)	-ve	-ve
Au/chitosan	+	+

Data represent mean ± SD of three independent experiments (*n* = 3). Statistical significance was determined by one-way ANOVA with Tukey’s post hoc test (*p* < 0.05). +: Positive.

## Data Availability

The original contributions presented in this study are included in the article. Further inquiries can be directed to the corresponding author.
